# Engineering of HIV-1 neutralizing antibody CAP256V2LS for manufacturability and improved half life

**DOI:** 10.1038/s41598-022-22435-2

**Published:** 2022-10-25

**Authors:** Baoshan Zhang, Deepika Gollapudi, Jason Gorman, Sijy O’Dell, Leland F. Damron, Krisha McKee, Mangaiarkarasi Asokan, Eun Sung Yang, Amarendra Pegu, Bob C. Lin, Cara W. Chao, Xuejun Chen, Lucio Gama, Vera B. Ivleva, William H. Law, Cuiping Liu, Mark K. Louder, Stephen D. Schmidt, Chen-Hsiang Shen, Wei Shi, Judith A. Stein, Michael S. Seaman, Adrian B. McDermott, Kevin Carlton, John R. Mascola, Peter D. Kwong, Q. Paula Lei, Nicole A. Doria-Rose

**Affiliations:** 1 Vaccine Research Center, National Institute of Allergy and Infectious Diseases, National Institutes of Health, 40 Convent Drive, Bethesda, MD 20892 USA; 2 Beth Israel Deaconess Medical Center, Boston, MA USA; 3 Harvard Medical School, Boston, MA USA

**Keywords:** Immunology, Proteins

## Abstract

The broadly neutralizing antibody (bNAb) CAP256-VRC26.25 has exceptional potency against HIV-1 and has been considered for clinical use. During the characterization and production of this bNAb, we observed several unusual features. First, the antibody appeared to adhere to pipette tips, requiring tips to be changed during serial dilution to accurately measure potency. Second, during production scale-up, proteolytic cleavage was discovered to target an extended heavy chain loop, which was attributed to a protease in spent medium from 2-week culture. To enable large scale production, we altered the site of cleavage via a single amino acid change, K100mA. The resultant antibody retained potency and breadth while avoiding protease cleavage. We also added the half-life extending mutation LS, which improved the in vivo persistence in animal models, but did not impact neutralization activity; we observed the same preservation of neutralization for bNAbs VRC01, N6, and PGDM1400 with LS on a 208-virus panel. The final engineered antibody, CAP256V2LS, retained the extraordinary neutralization potency of the parental antibody, had a favorable pharmacokinetic profile in animal models, and was negative in in vitro assessment of autoreactivity. CAP256V2LS has the requisite potency, developability and suitability for scale-up, allowing its advancement as a clinical candidate.

## Introduction

Broadly neutralizing antibodies (bNAbs) against HIV are potentially useful for prevention or treatment of infection^1^. The recent AMP clinical trial tested the effect of the bNAb VRC01 for prevention of infection; while there was no overall efficacy, VRC01 blocked infection of the most antibody-sensitive viruses, with 75% efficacy against viruses that are neutralized with an in vitro ID80 of less than 1 μg/ml^2^. This result suggests that bNAbs with greater breadth and potency than VRC01 might be efficacious for HIV prevention. In addition, to maximize the coverage of diverse HIV strains, and minimize the effect of escape mutations at single epitopes, it is likely that multiple bNAbs targeting different epitopes would need to be used in combination. CAP256-VRC26.25 is an exceptionally potent bNAb, and is particularly broad and potent against non-B clade virus strains^3^. A study of bNAbs assessed against viruses of clade C, the most common clade worldwide, found that the most favorable combinations of bNAbs included CAP256-VRC26.25^4^. Therefore, production of CAP256-VRC26.25 has been a priority.

While CAP256-VRC26.25 can be easily produced in HEK-Expi293F cell cultures grown for up to 6 days, as is commonly done in research laboratories, large-scale production of CAP256-VRC26.25 was hampered by proteolytic cleavage, also referred to as clipping, of the antibody when produced in CHO cell culture for 14–17 days. This cleavage was attributed to a residual protease present in the spent culture medium, which attacks an exposed lysine residue in the highly extended CDRH3 loop of the heavy chain^5^. Multiple process strategies were evaluated to minimize the proteolytic cleavage observed during cell culture^6,7^. These included (1) a fill-draw approach that resembled a repeated fed batch, where 75% of the bioreactor was harvested on day 10 and refilled with fresh media every 3 days (2) inclusion of protease inhibitors for serine proteases^8,9^ in the cell culture to effectively inhibit proteolytic activity and (3) alternating tangential flow (ATF)-Perfusion cell culture^10,11^, allowing continuous antibody harvest. All three approaches minimized CAP256 cleavage below 5%; however, due to risks associated with scaling and transferring these processes, product stability risks, and long-term development risks, it was determined that engineering out the proteolytic cleavage site was better suited for long term clinical product development.

Here, we show that a single amino acid change in the CDRH3 was sufficient to prevent cleavage of CAP256-VRC26.25 while retaining neutralization activity. Further addition of the half-life extending mutation, LS, improved the pharmacokinetic profile in model animals with no impact on neutralization. The resulting modified antibody, CAP256V2LS, was suitable for scale-up to 2000 liter cultures, allowing its advancement to a promising clinical candidate.

## Results

### Mutant screening

**Figure 1:**
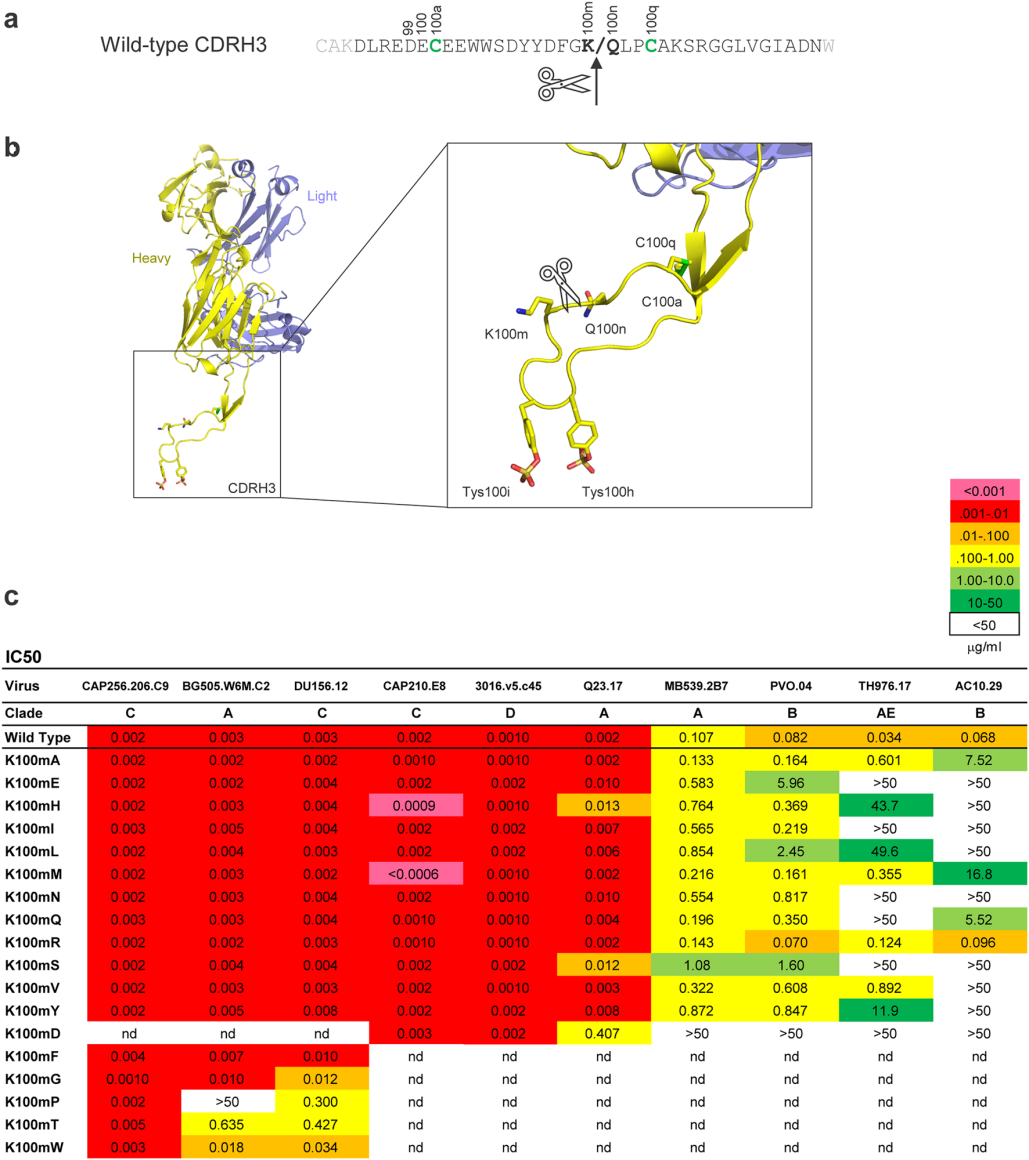
CAP256-VRC26.25 mutations at the proteolytic cleavage site have a variable effect on neutralization potency. (**a**) CDRH3 sequence of wild-type CAP256-VRC26.25. The arrow and slash indicate the site of cleavage. The positions are labeled according to the Kabat numbering scheme. Cysteines involved in a disulfide bond are highlighted in green. (**b**) The wild-type CAP256-VRC26.25 Fab is shown (PDB ID 5DT1) with the CDRH3 highlighted to show the location of the cleavage between K100m and Q100n and its relation to the disulfide bond (green) and sulfated tyrosines (stick representation). (**c**) Neutralization of Env-pseudovirus panel by CAP256.25 variants. Each Ab was expressed using the indicated CAP256-VRC26.25 heavy chain paired with wild type light chain. *nd* not done. Data are IC50 in μg/ml.

Previous work identified a site of proteolytic cleavage (clipping) at the lysine at position K100m (Kabat numbering) in the CAP256-VRC26.25 CDRH3^5^ (Fig. 1a,b). We therefore generated a series of mutants in CAP256-VRC26.25 heavy chain in which that position was changed to every possible residue except cysteine. The antibodies were tested against a panel of Env-pseudoviruses to check for loss of neutralization (Fig. 1c, Suppl Fig. S1A). Neutralization of Env-pseudovirus CAP256.209.c9 was unaffected by any of the mutations. Activity of most mutants against Env-pseudoviruses BG505 and DU156 was decreased five to 100-fold; the least affected mutants were further tested against MB539, TH976, PVO.04, and AC10.29. Mutation to alanine, methionine, or glutamine (K100mA, K100mM, and K100mQ) caused a small (< fivefold) overall reduction in potency, while the other mutations greatly reduced or abrogated activity. Changing the lysine to an arginine (K100mR) did not alter the neutralization activity. Of note, the CAP256-VRC26.25 appeared to be carried over in pipet tips during dilution, causing an artifact in which the neutralization activity does not titer out. This led us to adopt a neutralization assay protocol in which pipet tips are changed after each antibody dilution step; doing so was found to be critical for accurate assessment of CAP256-VRC26.25 neutralization potency (Suppl Fig. S2).

**Figure 2:**
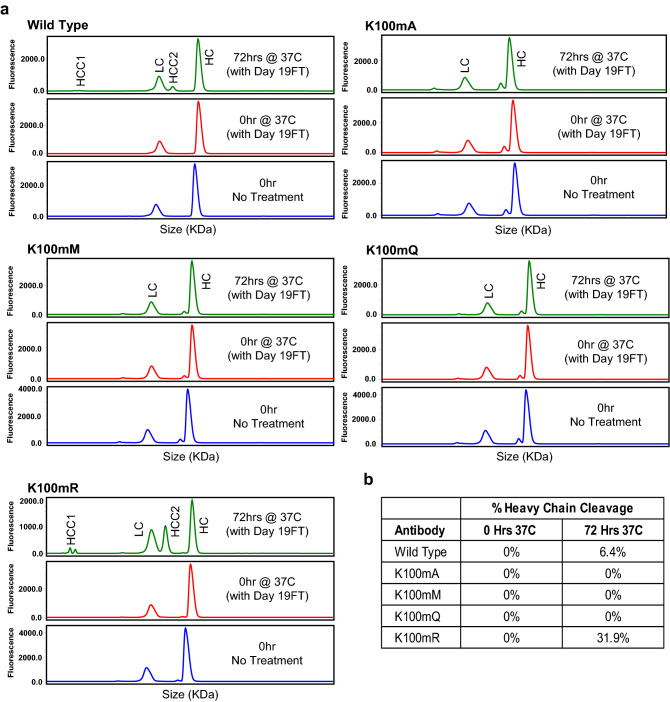
CAP256-VRC26.25 variants resist cleavage. (**a**) Samples were treated with day 19 CHO supernatant flow-through containing protease (Day 19FT) for 0 or 72 h. Treated and untreated samples were separated by size using reducing capillary gel electrophoresis. Peaks labelled HCC1, HCC2 contain clipped product; HC contains unclipped heavy chain, LC contains light chain. (**b**) % Cleaved heavy chain showing 0 and 72 h treatments. % HCC was calculated as area of each peak divided by total area of all peaks.

To mimic the exposure to proteases released by dead cells in 19-days CHO cell culture, we passed medium from 19 days CAP256-VRC26.25 CHO cell cultures over protein A and retained the flow-through fraction. The wild-type antibody and mutants K100mA, K100mM, K100mQ, and K100mR (produced in 6 days HEK-Expi293F cultures) were incubated in this protease-containing flow-through material at 37 °C for 0 or 72 h. After additional protein A column purification, treated or untreated mAbs were tested by reducing CGE-SDS (GX II) chromatography analysis to monitor heavy chain cleavage^12^. The chromatography trace for wild-type CAP256-VRC26.25 included extra peaks identified as the products of heavy-chain cleavage; 6.4% of the antibody mass was assigned to the clipped products (Fig. 2). We observed that the K100mR mutant was also subject to cleavage; this was not unexpected, as lysine and arginine are both targets of serine proteases, the presumptive class of proteases acting on the antibodies. In contrast, the K100mA, K100mM, and K100mQ variants were not cleaved (Fig. 2).

**Figure 3:**
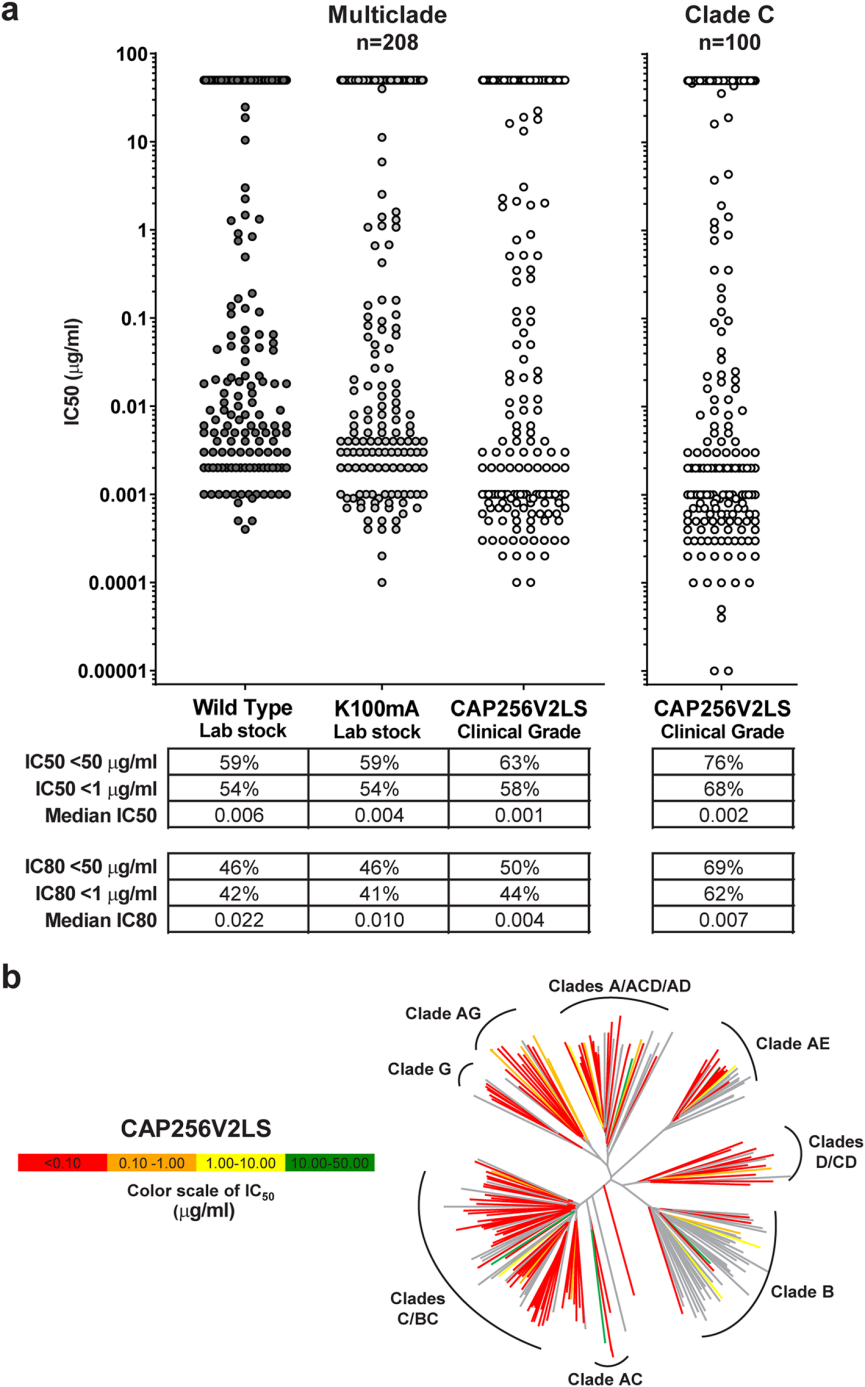
K100mA mutant maintains breadth and potency as assessed on large virus panels. (**a**) Neutralization by CAP256-VRC26.25 wild type, K100mA, and CAP256V2LS was measured against a multi-clade 208 Env-pseudovirus panel (left) and 100 acute Clade C Env-pseudoviruses (right). Each dot represents one pseudovirus. (**b**) Dendrogram shows phylogenetic relatedness of Env sequences in multi-clade panel, color-coded by neutralization sensitivity.

The cleavage–resistant variant that showed the greatest potency in the initial neutralization screen, K100mA, was further tested on a 208 multiclade Env-pseudovirus panel. When compared to wild type, this mutant had the same overall breadth and median potency (Fig. 3a, Table 1, Fig. S1, Table S1). At the level of individual viruses, we observed that K100mA gained potency for some viruses and lost potency for others, but in a balanced manner such that the total fraction of viruses sensitive to K100mA was the same as to wild type (Fig. S1B). We noted a very high correlation between IC50 values for the two antibodies (Spearman’s rho 0.96, p < 0.0001) as well as for IC80 values (Spearman’s rho 0.97, p < 0.0001). In addition, we used the GPS-TSP algorithm^13^ to predict the impact of K100mA on tyrosine sulfation of the CDRH3, as this post-translational modification is critical for full potency of the antibody (Cai et al., manuscript submitted). Predicted sulfation of wild-type and K100mA were nearly identical (Fig. S1C), in agreement with the observed retention of neutralization activity. Because the K100mA mutant retained the full breadth and potency of the wild type, while resisting proteolytic cleavage, we selected this variant for further development.

### Half-life extending mutation in lead variant

In addition to the K100mA mutation, we added the LS mutation in the Fc portion of the heavy chain, which was previously shown to increase the half-life of parental CAP256-VRC26.25 upon passive administration in non-human primates^14^. In humans, the LS mutations in other antibodies have been shown to increase serum half-life in vivo; the mechanism is related to increased antibody binding to the FcRn receptor^15,16^. LS is not expected to affect neutralization potency. To verify this, we assessed neutralization on the 208 multiclade Env-pseudovirus panel for three pairs of bNAbs: VRC01 and VRC01LS, N6 and N6-LS, and PGDM1400 and PGDM1400LS (Suppl Table S1E). As expected, only very minor differences were noted between the antibodies with and without LS (Table 1, Suppl Table S1A,B), with highly significant correlation between values: for each pair of antibodies, both IC50 values and IC80 values correlated with Spearman’s rho of 0.94 or higher and p < 0.0001; and the median IC50s were no more than twofold different, which is within experimental error for this assay^17^. We therefore proceeded to add LS to the Fc portion of the CAP256-VRC26.25.K100mA construct.

**Table 1:** Neutralization breadth and potency of monoclonal antibodies on a multiclade panel of 208 Env-pseudoviruses.

Antibody	% Viruses neutralized		Median IC50	% Viruses neutralized		Median IC80
	IC50 < 50 µg/ml	IC50 < 1.0 µg/ml		IC80 < 50ug/ml	IC80 < 1.0ug/ml	
CAP256-VRC26.25	59	54	0.006	46	42	0.022
CAP256-VRC26.25.K100mA	59	54	0.004	46	41	0.010
CAP256V2LS	63	58	0.001	50	44	0.004
PGDM1400	80	75	0.014	74	63	0.047
PGDM1400-LS	79	73	0.015	74	62	0.058
10–1074	63	60	0.054	60	52	0.126
PGT121	65	56	0.039	59	49	0.099
PGT121.414.LS	58	50	0.022	50	45	0.051
3BNC117	85	77	0.109	80	68	0.298
N6	97	94	0.086	96	86	0.238
N6-LS	98	95	0.069	97	88	0.221
VRC01	90	72	0.328	89	46	0.959
VRC01LS	90	76	0.260	88	52	0.704
VRC07-523-LS	96	92	0.081	96	83	0.238
CAP256V2LS + VRC07-523-LS	98	97	0.012	98	90	0.071

The resulting construct, CAP256V2LS, was produced at high purity under GMP conditions. This clinical-grade product was assessed for neutralization on the 208 multiclade Env-pseudovirus panel and was slightly more potent and broad than research lots of either wild type or K100mA (Fig. 3, Suppl Fig. S3, Table 1, Suppl Table S1A,B). This improvement may be due to enhanced purity of the clinical-grade product compared to research laboratory stocks, and/or improved tyrosine sulfation in the CHO cell line used for production^18^. Overall, clinical-grade CAP256V2LS is 63% broad on 208 viruses, with high potency and 70% breadth across non-B clade viruses, at a cutoff of IC50 < 50 μg/ml. At the more stringent cutoff of ID80 < 1 μg/ml suggested by the AMP trial results, breadth on non-clade B was 44% overall and 59% against clade C pseudoviruses in the panel (Fig. 3, Table S1A,B). The potency of CAP256V2LS is greater than other HIV-1 bNAbs that have been in clinical trials (Table S1A,B); and its breadth, while lower than some of them, is similar to mAb 10–1074. GMP-grade CAP256V2LS was also tested on a panel of 100 clade C viruses, representing the dominant sequences in southern Africa, with 62% breadth at ID80 < 1 and a median IC50 of 0.002 and ID80 of 0.007 μg/ml (Fig. 3, Suppl Table S1C,D). The combination with VRC07-523-LS had a predicted breadth of 90% at ID80 < 1 on both the multiclade and the clade C panels (Table 1, Suppl Table S1B,D).

### Structural analysis of mutant

**Figure 4:**
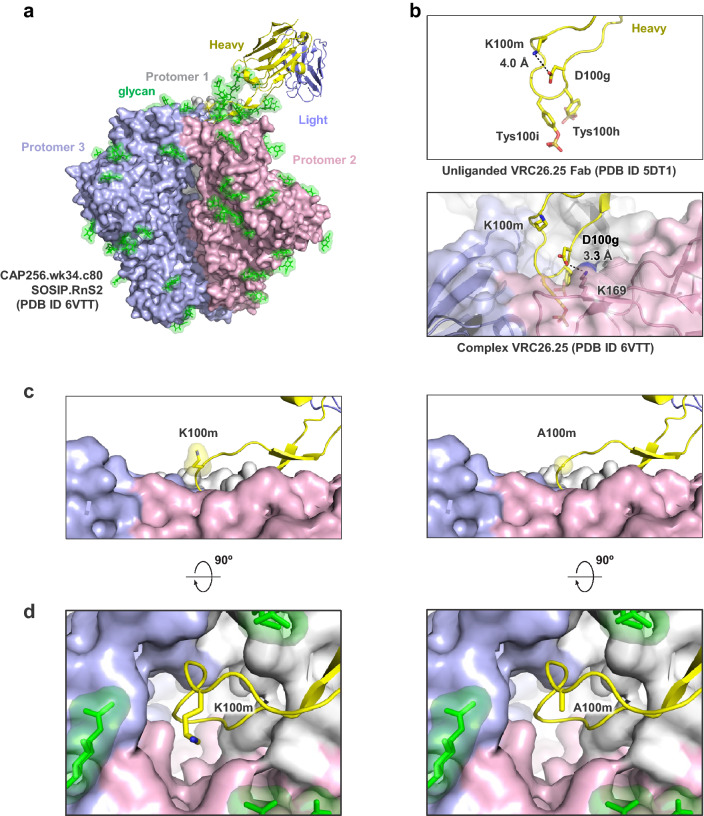
Position 100m in the CAP256-VRC26.25 CDRH3 does not contact the HIV-1 Env. (**a**) Cryo-EM structure of CAP256-VRC26.25 in complex with the CAP256.wk34.c80 SOSIP.RnS2 Env trimer. Trimer is shown in surface rendering, with protomers in grey, pink, and light blue; glycans in green; antibody heavy chain in yellow ribbon, light chain in slate ribbon. (**b**) Close-up of the unliganded CDRH3 conformation, highlighting the K100m contact with D100g (top) in contrast to the bound Fab where D100g is making electrostatic contacts with K169 of the Env. (**c**) (left) K100m is located above the central cavity in the Env apex facing the solvent. (right) modeled alanine at position 100m indicates no addition or loss of Env contacts. (**d**) The central cavity relative to position 100m is shown at a 90° rotation from (**c**).

To understand why the K100mA mutation had only minor effects on neutralization breadth and potency, we examined the structure of wild-type CAP256-VRC26.25 alone and in complex with a closed prefusion Env trimer (Fig. 4a,b). In the unliganded Fab structure^3^, K100m formed an electrostatic interaction with D100g of the CDRH3, while in the bound structure^19^ the D100g residue of the CDRH3 interacted with the positively charged K169 residue of the HIV-1 Env, thereby breaking contact with K100m (Fig. 4b). The location and orientation of the wild-type K100m outside of the apex cavity and facing the solvent prevents any meaningful contact with the Env trimer beyond minimal buried surface area of under 10 Å^2^ on the backbone. The side chain faced away from the Env apex cavity and away from any potential Env interactions. As expected from this observation, modelling of Ala at position 100 m onto the cryo-EM structure resulted in no change in the Env binding interactions (Fig. 4c,d).

### Pharmacokinetic and autoreactivity analyses

**Figure 5:**
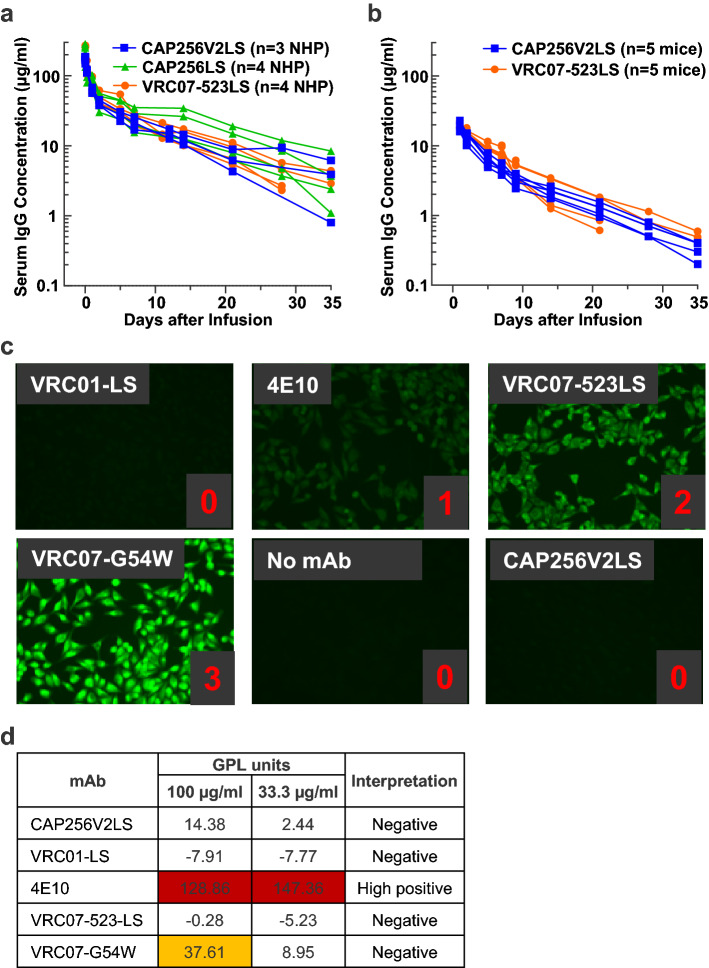
CAP256V2LS has a favorable pharmacokinetic (PK) profile in animal models and no autoreactivity in vitro. (**a**) PK in rhesus macaques (non-human primates, NHP). Naïve rhesus macaques were infused with 10 mg/kg of antibody, and the antibody concentrations were measured by an anti-idiotype based ELISA. Each line represents one animal. (**b**) PK in human FcRn mice. Naïve human FcRn mice were infused with 5 mg/kg of CAP256V2LS or VRC07-523LS and antibody concentrations were measured by an anti-idiotype based ELISA. (**c**) Hep-2 cell staining. (**d**) Cardiolipin binding at 100 and 33 μg/ml inputs; values shown as GPL units. VRC01-LS, 4E10, VRC07-523-LS, and VRC07-G54W are included as controls in (**c,d**).

Since CAP256V2LS is of interest for clinical use in HIV prevention, we tested its pharmacokinetic (pK) profile in rhesus macaques and in FcRn mice. The latter are transgenic mice expressing the human FcRn receptor and are commonly used to assess serum half-life of monoclonal antibodies^20^. Rhesus macaques were infused with 10 mg/kg of CAP256V2LS and parental CAP256-VRC26.25 with LS (CAP256LS); antibody concentrations above 10 μg/ml were maintained for three weeks post-infusion for both constructs (Fig. 5a). This is similar to published values for VRC07-523LS, which is a potent CD4 binding site antibody that is under clinical investigation for both HIV-1 prevention and therapy^21^. Concordant data were observed in a second model, human FcRn transgenic mice, in which CAP256V2LS had pK curves similar to VRC07-523LS (Fig. 5b). We also tested for autoreactivity in vitro, which can be predictive of poor pharmacokinetic properties in vivo^22^. CAP256V2LS was negative in cardiolipin binding and Hep-2 cell staining, indicating a lack of autoreactivity (Fig. 5c,d). We did not observe any anti-drug activity (ADA) in the rhesus pK studies.

## Discussion

Proteolytic cleavage during large-scale production of antibodies can cause severe loss of product and reduced potency, a major roadblock to clinical advancement^23^. We found a single mutation in CAP256-VRC26.25 that conferred both resistance to cleavage and retention of neutralization activity. The K100mA mutation allows production at large scale of this highly potent bNAb. This mutation was paired with the LS mutation in Fc, and the resulting CAP256V2LS antibody has a pharmacokinetic profile in NHP and FcRn mice similar to VRC07-523LS, which has a half-life of about 40 days in humans^24^. CAP256V2LS was negative in tests for autoreactivity, also a favorable finding for clinical use^22^.

Modelling of the K100mA mutation onto the cryo-EM structure suggests that the amino acid at this position has minimal contacts with the Env trimer, explaining the retention of full activity. It is also possible that there are transient interactions with the mobile N160 glycans in the region, although no contacts were observed in the structure with the native K100m. Despite the lack of contact with trimer at this position, certain amino acids did reduce neutralization activity. Indirect structural effects may explain two of the mutations that had the greatest impact on neutralization: glycine and proline at K100m may affect the peptide backbone flexibility, and thus may reduce the ability of the CDRH3 loop to insert into the trimer apex cavity.

The discovery of a single mutation that mitigates the proteolytic cleavage while retaining neutralization breadth and potency has allowed large-scale GMP production of the clinical candidate CAP256V2LS to proceed. This antibody is undergoing clinical testing for safety and pharmacokinetics in humans^25^, alone and in combination with VRC07-523-LS (Table S1). CAP256V2LS has the potential to be a valuable weapon in the fight against the HIV-1 pandemic.

## Methods

### Cleavage monitoring and quantification

To model the conditions of proteolytic cleavage in 19 days cell culture, we first generated protease-containing medium as follows: CHO cells transfected with CAP256V2LS plasmids were grown for 19 days in a Fed-batch bioreactor and harvested on 19th day. The harvest was subjected to protein A column to remove the antibody, and flow through cell culture media was collected and saved as day 19 cell culture harvest supernatant (Day19FT), which contains active protease. To assess the level of cleavage, 0.5 ml of 2 mg/ml antibody sample was incubated with 1.5 ml of Day19FT at 37 °C for 72 h, or immediately chilled at 4 °C (0 h) as a control; and then purified using small ProA columns. The purified mAbs were then tested on GX II under reducing conditions to monitor % heavy chain cleavage^12^. For GXII assay, 8ug of protein was treated with 30 mM DTT (reducing) in sample buffer containing SDS and were denatured and reduced at 90 °C for 5 min. Protein was then subjected to capillary gel electrophoresis (CGE-SDS) and results were analyzed using Perkin Elmer GXII software. %Heavy chain cleaved peaks were calculated by relative peak area of cleaved peaks to total peak area of heavy and light chain in the reduced sample.

### Mutagenesis and protein production

Antibody heavy chain and light chain expression constructs were generated through site-directed mutagenesis (GeneImmune Biotechnology). The antibody variants were expressed by transient transfection in HEK-Expi293F cells (Thermo Fisher) using Turbo293 transfection reagent (SPEED BioSystems) according to the manufacturer’s recommendation. 25 µg plasmid encoding heavy-chain and 25 µg plasmid encoding light-chain variant genes were mixed with 150 ml transfection reagents, added to 50 ml of Expi293 cells at 2.5 × 10^6^ per microliter, and incubated in a shaker incubator at 120 rpm, 37 °C, 9% CO_2_. At 5 days post-transfection, cell culture supernatant was harvested and purified with a Protein A (GE Healthcare) column. The antibodies were eluted using IgG Elution Buffer (Thermo Fisher) and were brought to neutral pH with 1 M Tris–HCl, pH 8.0. Eluted antibodies were dialyzed against PBS overnight and were evaluated by SDS-PAGE before use.

### 50 liter production to clinical GMP production

The CAP256V2LS fed-batch process was developed at the 50 L scale and successfully scaled-up to a 2000 L bioreactor for GMP production of clinical trial material using chemically defined, animal component-free medium. Briefly, one vial of the master cell bank is thawed in shake flasks, expanded to a 250 L Single-use Bioreactor (SUB) prior to inoculation of the 2000 L production bioreactor. Purification included a combination of depth filtration, three chromatography steps, viral inactivation and filtration, diafiltration, formulation and concentration. Analytical testing assured product quality. CAP256V2LS was produced from a single cell clone that ranged in productivity from 2.4 to 2.9 g/L titers in clinical material production runs.

### mAb neutralization

Neutralization was assessed in one of three formats of the Env-pseudotyped assay^17^, all of which yield highly similar results.

1. Screening of variants was performed in 96-well format as follows: 10 μl of fivefold serially diluted mAbs in cDMEM was incubated with 40 μl of diluted HIV-1 Env-pseudotyped virus and incubated for 30 min at 37 °C in a 96-well CulturPlate (Perkin Elmer). 20 μl of TZM-bl cells (10,000 cells/well) with or without 70 μg/ml DEAE-Dextran was then added and incubated overnight at 37 °C. Each experiment plate also had a column of cells only (no Ab or virus) and a column of virus only (no Ab) as controls for background TZM-bl luciferase activity and maximal viral entry, respectively. Serial dilutions were performed with a change of tips at each dilution step to prevent carryover; the importance of this change is illustrated in Fig. S2. The following day, all wells received 100 μl of fresh cDMEM and were incubated overnight at 37 °C. The following day, 50 μl of Steadylite Plus Reporter Gene Assay System (PerkinElmer) was added to all wells, and plates were shaken at 600RPM for 15 min. Luminometry was then performed on a SpectraMax L (Molecular Devices) luminometer. Percent neutralization is determined by calculating the difference in average Relative Light Units (RLU) between virus only wells (cells + virus column) and test wells (cells + plasma/Ab sample + virus), dividing this result by the average RLU of virus only wells (cell + virus column) and multiplying by 100. Background is subtracted from all test wells using the average RLU from the uninfected control wells (cells only column) before calculating the percent neutralization. Neutralizing plasma antibody titers are expressed as the antibody concentration required to achieve 50% neutralization and calculated using a dose–response curve fit with a 5-parameter nonlinear function.
2. Select monoclonal antibodies were assessed on a panel of 208 geographically and genetically diverse Env pseudoviruses representing the major subtypes and circulating recombinant forms^26^. Assays were performed by microneutralization in an optimized and qualified automated 384-well format as described^17^, with the addition of changing tips after each antibody dilution. Data were analyzed as above.
3. CAP256V2LS was assessed on a panel of 100 clade C Env pseudoviruses from acute infection^4^. Neutralization assays were conducted using TZM.bl cells as previously described^17^. Briefly, mAb samples were tested in duplicate in 96-well plates using a primary concentration of 50 μg/ml or 1 μg/ml and serially diluted fivefold seven times. The 1 μg/ml start value was used instead of changing tips, as noted above. HIV-1 Env pseudovirus was added to antibody serial dilutions and plates were incubated for 1 h at 37 °C. TZM.bl cells were then added at 1 × 10^4^/well with DEAE-Dextran at a final concentration of 11 μg/ml. After 48 h incubation at 37 °C, plates were harvested using Promega Bright-Glo luciferase (Madison, WI) and luminescence detected using a Promega GloMax Navigator luminometer. Antibody concentrations that inhibited 50% or 80% of viral infection were determined (IC50 and IC80 titers, respectively). Neutralization assays were conducted in a laboratory meeting Good Clinical Laboratory Practice quality assurance criteria.

### Modeling

PDB ID 5DT1 was used for the unliganded CAP256-VRC26.25 Fab and PDB ID 6VTT was used for the CAP256-VRC26.25 Env complex structure. Mutations were made using Coot v0.9 and figures made using Pymol v2.4.2. Refinement, simulated annealing, and MDFF were performed with the mutated coordinates with density from the WT structure using Phenix v1.19 and Isolde v1.1 in ChimeraX v1.1.1 with no significant change in the position of A100m compared to the WT K100m. The trimer in the complex, CAP256.wk34.c80 SOSIP.RnS2, has a sequence derived from an Env clone from the CAP256 donor^19^ likely to have initiated the CAP256-VRC26 lineage^27^.

### Tyrosine sulfation prediction

Tyrosine sulfation within the CDRH3 was predicted from the amino acid sequence using GPS-TSP software available at http://tsp.biocuckoo.org/^13^.

### Autoreactivity

Autoreactivity was determined by ANA Hep-2 Staining Analysis (ZEUS Scientific Cat. No: FA2400) and anticardiolipin ELISA (Inova Diagnostics Cat. No.: 708625). For the Hep-2 assay, all antibodies were tested at 25 and 50 μg/ml as per manufacturer’s protocol and imaged on a Nikon Ts2R microscope for 500 ms. Scores from 0 to 3 were defined with four control antibodies VRC01-LS, 4E10, VRC07-523LS, and VRC07-G54W. Test antibodies were scored by visual estimation of staining intensity in comparison to the control antibodies. Scores equal to or greater than 1 at 25 μg/ml were classified as autoreactive, and between 0 and 1 as mildly autoreactive. In the cardiolipin ELISA, antibodies were tested at a starting concentration of 100 μg/ml, followed by threefold dilutions. IgG phospholipid (GPL) units were calculated from the standard curve. GPL score < 20 was considered as not reactive, 20–80 as low positive and > 80 as high positive.

### Pharmacokinetic studies in NHPs and human FcRn mice

Eleven rhesus macaques of Indian origin (Macaca mulatta) comprising of seven male and four female animals with mean age of 7.2 ± 5.2 years and mean weight of 6.5 ± 3.3 kg were used in this study. Out of these animals, four male animals were infused with VRC07-523LS; two male and two female animals with CAP256LS; and one male and two female animals with CAP256V2LS. All infusions were done intravenously with a 10 mg/kg dose for each mAb. Endotoxin levels were measured for each antibody preparation by the QCL-1000™ endpoint chromogenic LAL assay (Lonza) and were all below 0.5 EU/mg levels. Serum samples were collected prior to injection, and at time points 0, 30 min, 6 h, 12 h, and days 1, 2, 4, 7, 14, 21, 28 and 35. All the sampling, weighing and infusion procedures were done on anaesthetized animals using either ketamine or ketamine/dexmedetomidine cocktail as the anaesthesia agent. Serum was separated by centrifugation. Serum samples were heat inactivated at 56 °C for 30 min, and lipoproteins were pelleted.

Serum mAb levels were determined by an anti-idiotype based ELISA as described previously^21^. All animals were housed and cared for in accordance with Guide for Care and Use of Laboratory Animals Report number NIH 82-53 (Department of Health and Human Services, Bethesda, Maryland, USA, 1985) in a biosafety level 2 National Institute of Allergy and Infectious Diseases (NIAID) facility. All animal procedures and experiments were performed according to protocols approved by the Institutional Animal Care and Use Committee of the National Institute of Allergy and Infectious Diseases (NIH).

Male human FcRn transgenic mice (C57BL/6, B6.mFcRn−/−hFCRN Tg32 line from The Jackson Laboratory) with mean age of 10.9 ± 1.2 weeks and mean weight of 22.2 ± 3.2 g were used to assess the pharmacokinetics of CAP256V2LS and VRC07-523LS antibodies. Each animal was infused intravenously with 5 mg mAb/kg of body weight. Whole blood samples were collected at day 1, 2, 5, 7, 9, 14, 21, 28 and 35. Serum was separated by centrifugation. Serum mAb levels were measured by an anti-idiotype based ELISA as described previously^21^. All mice were bred and maintained under pathogen-free conditions at an American Association for the Accreditation of Laboratory Animal Care (AAALAC)-accredited animal facility at the NIAID and housed in accordance with the procedures outlined in the Guide for the Care and Use of Laboratory Animals. All mice were between 6 and 13 weeks of age. The study protocol was evaluated and approved by the NIH Animal Care and Use Committee (ASP VRC-18-747). This study is reported in accordance with ARRIVE guidelines.

## Supplementary Information

Supplementary Figures.

Supplementary Table S1.

## Data Availability

Neutralization data as reported in Table S1A–D are also available through the CATNAP database, https://www.hiv.lanl.gov/components/sequence/HIV/neutralization/.
