# Supplementary material for: Engineering of HIV-1 neutralizing antibody CAP256V2LS for manufacturability and improved half life
Source: Sci Rep. 2022 Oct 25;12:17876. doi: 10.1038/s41598-022-22435-2 (PMC9596707; doi:10.1038/s41598-022-22435-2)
Supplement: Supplementary file 1 — Supplementary Figures. [file 41598_2022_22435_MOESM1_ESM.pdf]

Supplemental materials

Engineering of HIV-1 neutralizing antibody CAP256V2LS for manufacturability and improved half life

Baoshan Zhang[1], Deepika Gollapudi[1], Jason Gorman[1], Sijy O'Dell[1], Leland F. Damron[1], Krisha McKee[1], Mangaiarkarasi Asokan[1], Eun Sung Yang[1], Amarendra Pegu[1], Bob C. Lin[1], Cara W. Chao[1], Xuejun Chen[1], Lucio Gama[1], Vera B. Ivleva[1], William H. Law[1], Cuiping Liu[1], Mark K. Louder[1], Stephen D. Schmidt[1], Chen-Hsiang Shen[1], Wei Shi[1], Judith A. Stein[1], Michael S. Seaman[2,3], Adrian B. McDermott[1], Kevin Carlton[1], John R. Mascola[1], Peter D. Kwong[1], Q. Paula Lei[1], Nicole A. Doria-Rose[1]

[1]Vaccine Research Center, National Institute of Allergy and Infectious Diseases, National Institutes of Health, Bethesda MD, USA

[2]Beth Israel Deaconess Medical Center, Boston, MA, USA

[3]Harvard Medical School, Boston, MA, USA

a

IC80

	CAP256.206.C9	BG505.W6M.C2	DU156.12	CAP210.E8	3016.v5.c45	Q23.17	MB539.2B7	PVO.04	TH976.17	AC10.29
**Clade**	C	A	C	C	D	A	A	B	AE	B
**Wild Type**	0.006	0.013	0.020	0.005	0.004	0.185	1.87	0.586	>50	3.94
**K100mA**	0.005	0.008	0.014	0.003	0.003	0.153	2.75	1.94	>50	>50
**K100mE**	0.005	0.021	0.039	0.007	0.007	24.6	>50	>50	>50	>50
**K100mH**	0.005	0.030	0.063	0.004	0.004	12.9	>50	9.36	>50	>50
**K100mI**	0.008	0.031	0.042	0.005	0.007	3.16	>50	>50	>50	>50
**K100mL**	0.005	0.024	0.027	0.005	0.006	5.48	>50	>50	>50	>50
**K100mM**	0.006	0.013	0.014	0.004	0.004	0.198	6.17	2.77	>50	>50
**K100mN**	0.005	0.034	0.048	0.004	0.005	5.39	>50	16.6	>50	>50
**K100mQ**	0.008	0.019	0.032	0.005	0.005	0.437	6.02	6.11	>50	>50
**K100mR**	0.006	0.015	0.022	0.004	0.005	0.193	2.71	0.567	>50	16.4
**K100mS**	0.005	0.036	0.048	0.005	0.008	>50	>50	48.8	>50	>50
**K100mV**	0.006	0.012	0.030	0.005	0.004	1.09	10.6	11.8	>50	>50
**K100mY**	0.007	0.070	0.074	0.007	0.006	24.1	>50	>50	>50	>50
**K100mD**	nd	nd	nd	0.006	0.011	>50	>50	>50	>50	>50
**K100mF**	0.011	0.091	0.208	nd	nd	nd	nd	nd	nd	nd
**K100mG**	0.004	0.838	0.241	nd	nd	nd	nd	nd	nd	nd
**K100mP**	0.006	>50	>50	nd	nd	nd	nd	nd	nd	nd
**K100mT**	0.013	>50	19.2	nd	nd	nd	nd	nd	nd	nd
**K100mW**	0.008	0.786	0.854	nd	nd	nd	nd	nd	nd	nd

<0.001
.001-.01
.01-.100
.100-1.00
1.00-10.0
10-50
<50
µg/ml

b

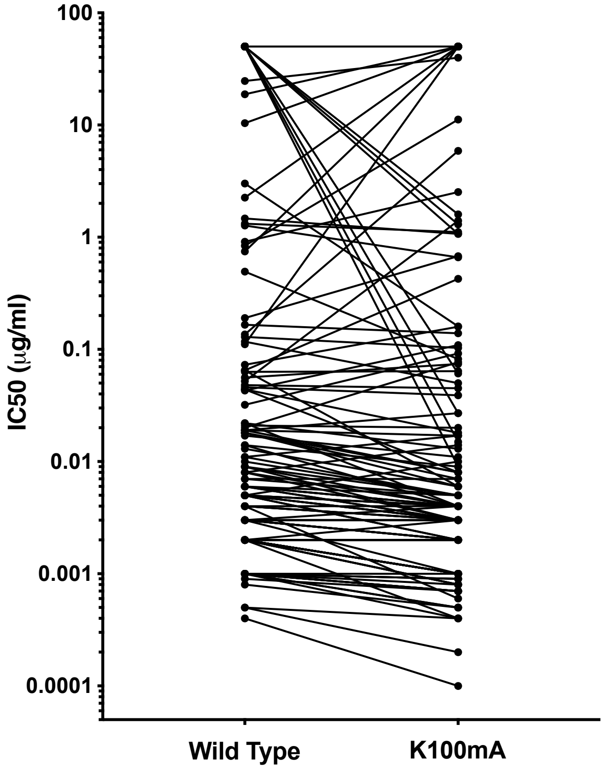

C

Variant	Peptide	Score
Wild Type	CEEWWSDYYDFGKQL	1.168
	EEWWSDYYDFGKQLP	1.827
K100mA	CEEWWSDYYDFGAQL	1.183
	EEWWSDYYDFGAQLP	1.802

CAKDLREDECEEWWSDYYDFGKQLPCAKSRGGLVGIADNW

100h 100i

Figure S1. Neutralization of Env-pseudovirus panel and predicted tyrosine sulfation by CAP256.25 variants. **A.** Each Ab was expressed using the indicated CAP256-VRC26.25 heavy chain paired with wild type light chain. nd, not done. Data are IC80 in µg/ml. **B.** Comparison of wild type and K100mA on 208 Env-pseudoviruses. Each pair of dots, connected by a line, shows the IC50 against the same pseudovirus. **C.** Prediction of tyrosine sulfation by GPS-TSP algorithm. Scores above 1.139 predict sulfation, and refer to amino acid in red. Each peptide is a 15-mer centered on the tyrosine under analysis.

a

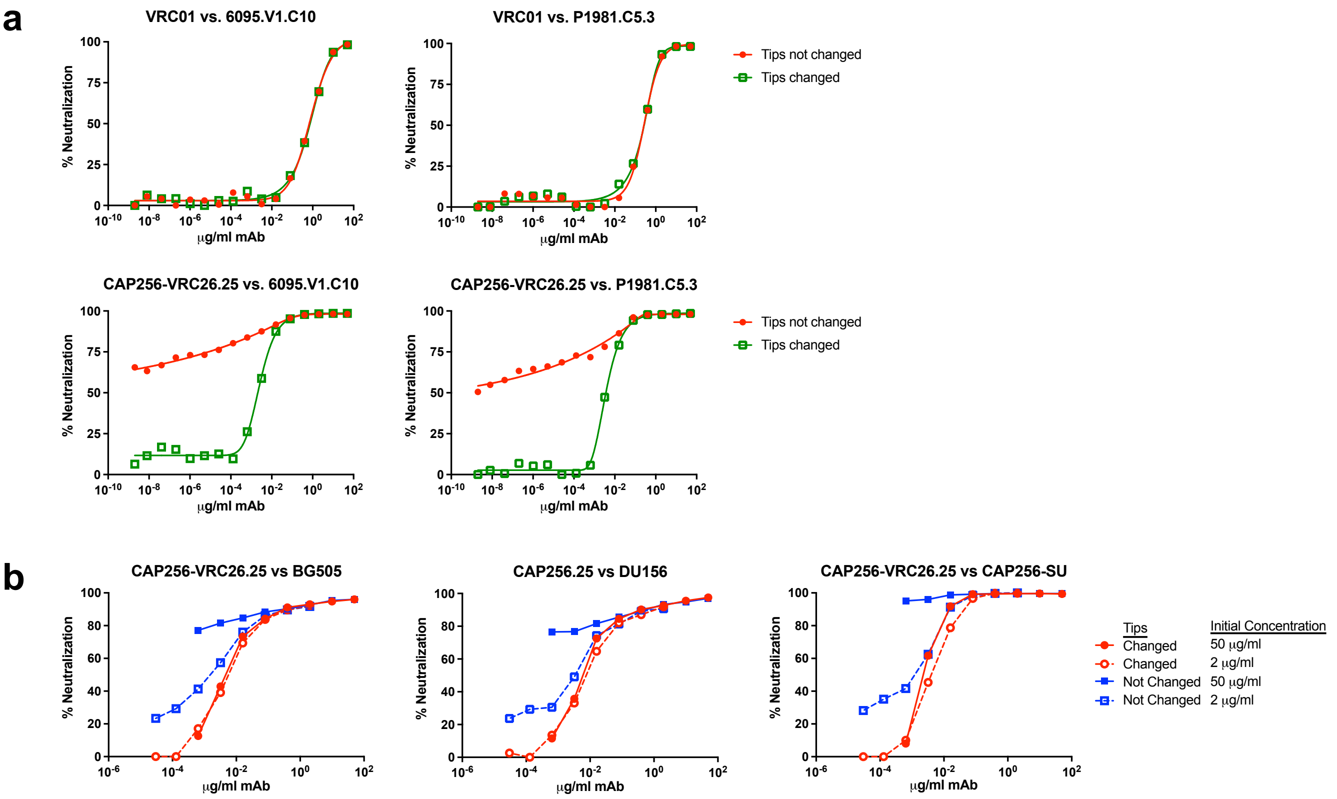

c

IC50		CAP256-VRC26.25		10E8		VRC01		10E8-V5R-100cF	
	Clade	No Tip Change	Tip Change	No Tip Change	Tip Change	No Tip Change	Tip Change	No Tip Change	Tip Change
6101.1	B	>50	>50	0.001	0.004	0.079	0.056	<0.000000002	0.002
0013095-2.11	C	7.94	>50	0.016	0.034	0.24	0.304	<0.000000002	0.004
3326.V4.C3	CD	<0.000000002	0.001	1.24	1.39	0.158	0.15	0.039	0.097
6095.V1.C10	ACD	<0.000000002	0.003	0.002	0.006	0.87	1.12	<0.000000002	0.0009
BG505.W6M.C2	A	<0.000000002	0.002	0.391	0.47	0.058	0.066	0.033	0.074
CAP256.206.C9	C	<0.000000002	0.002	0.655	0.841	0.719	0.899	0.068	0.138
CNE40	BC	0.035	0.108	0.002	0.005	0.517	0.508	<0.000000002	0.001
CNE59	AE	>50	>50	0.005	0.007	0.635	0.768	<0.000000002	0.001
DJ263.8	AG	<0.000000002	0.005	0.012	0.024	0.111	0.092	<0.000000002	0.003
DU156.12	C	0.000000008	0.005	0.013	0.018	0.12	0.099	<0.000000002	0.002
MW965.26	C	0.004	0.038	0.002	0.004	0.037	0.041	<0.000000002	0.0006
P1981.C5.3	G	<0.000000002	0.005	0.031	0.033	0.355	0.324	0.0000005	0.005
ZM53.12	C	<0.000000002	0.002	4.69	3.79	0.772	1.03	0.307	0.389

IC80		CAP256-VRC26.25		10E8		VRC01		10E8-V5R-100cF	
	Clade	No Tip Change	Tip Change	No Tip Change	Tip Change	No Tip Change	Tip Change	No Tip Change	Tip Change
0013095-2.11	C	>50	>50	0.09	0.113	0.484	0.482	0.0005	0.016
3326.V4.C3	CD	<0.000000002	0.004	3.9	3.82	8.26	3.97	0.306	0.334
6095.V1.C10	ACD	0.0000002	0.008	0.014	0.023	2.66	2.69	<0.000000002	0.003
6101.1	B	>50	>50	0.017	0.021	0.172	0.161	0.00002	0.012
BG505.W6M.C2	A	0.0001	0.012	1.79	1.72	0.184	0.152	0.174	0.27
CAP256.206.C9	C	0.00000003	0.006	2.78	3.6	2.25	2.22	0.384	0.431
CNE40	BC	0.813	1.02	0.012	0.017	3.92	3.85	<0.000000002	0.004
CNE59	AE	>50	>50	0.024	0.026	2.37	2.17	<0.000000002	0.004
DJ263.8	AG	0.0003	0.015	0.103	0.113	0.759	0.477	0.0002	0.014
DU156.12	C	0.039	0.059	0.058	0.095	0.332	0.276	0.00005	0.012
MW965.26	C	0.141	0.421	0.007	0.013	0.104	0.112	<0.000000002	0.002
P1981.C5.3	G	0.001	0.019	0.105	0.122	0.821	0.723	0.0008	0.016
ZM53.12	C	<0.000000002	0.005	10.9	9.58	3.49	3.84	0.936	0.882

<0.001
.001-.01
.01-.100
.100-1.00
1.00-10.0
10-50
<50
μg/ml

Figure S2. Changing pipet tips during antibody dilution dramatically improves accuracy of CAP256-VRC26.25 neutralization assays. A. Representative neutralization curves for CAP256-VRC26.25 and VRC01. Assays were performed manually, with or without changing tips during 16 five-fold antibody dilutions. When tips are not changed, the activity of CAP256-VRC26.25 does not titer out. With tip change, the typical sigmoidal curve is observed. **B**. CAP256-VRC26.25 was assayed manually at a starting concentration of 50 or 2 μg/ml, with or without changing tips, with 8 five-fold antibody dilutions . In the absence of changing tips, starting at a lower concentration brings curves closer to the expected sigmoidal shape, but the antibody does not completely titer out. **C.** IC50 and IC80 for four antibodies using 384 well format assay with robotic pipetting, with or without changing tips during 16 five-fold antibody dilutions. 10E8-V5R-100cF and CAP256-VRC26.25 are highly potent bNAbs.

a

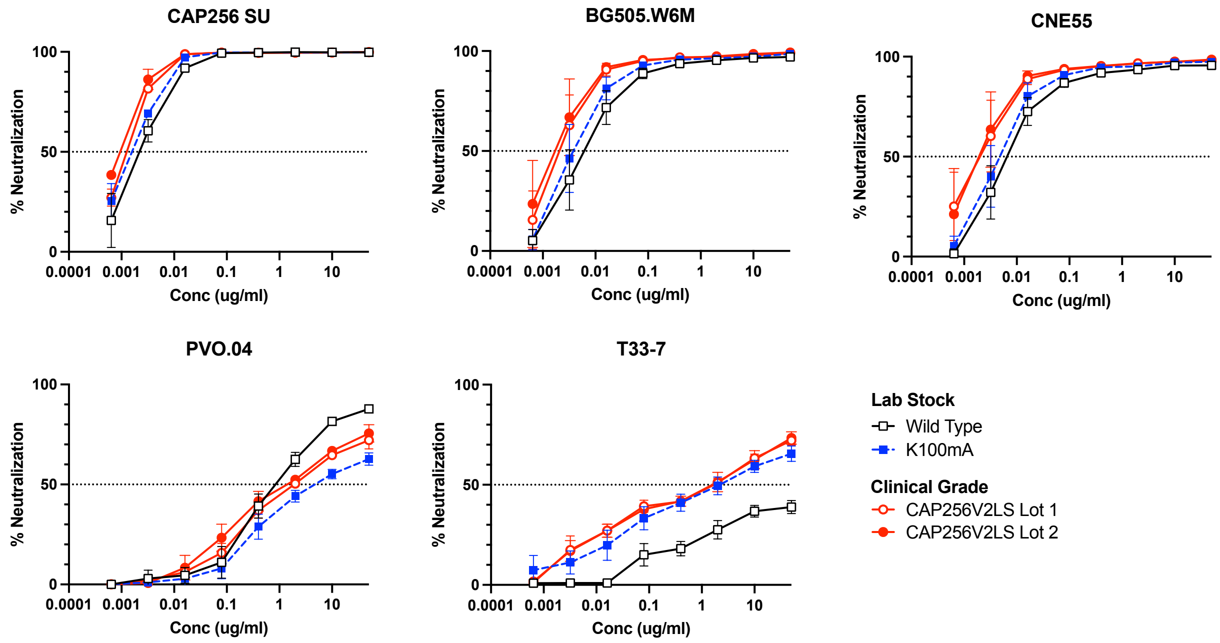

b

IC50		Lab stock (HEK 293expi)		Clinical-grade (CHO)	
Virus	Clade	CAP256-VRC26.25	CAP256-VRC26.25.K100mA	CAP256V2LS lot 1	CAP256V2LS lot 2
3016.v5.c45	D	0.005	0.004	0.002	0.002
AC10.29	B	1.42	>50	26.3	44.6
BG505.W6M.C2	A	0.006	0.004	0.002	0.002
CAP210.E8	C	0.007	0.005	0.002	0.003
CAP256.206.C9	C	0.002	0.002	0.0009	0.0007
CNE55	AE	0.006	0.004	0.002	0.002
KER2018.11	A	0.004	0.003	0.001	0.001
PVO.04	B	0.783	3.56	1.07	1.59
T33-7	AG	>50	1.62	0.946	0.854
ZM53.12	C	0.006	0.005	0.002	0.003

IC80		Lab stock (HEK 293expi)		Clinical-grade (CHO)	
Virus	Clade	CAP256-VRC26.25	CAP256-VRC26.25.K100mA	CAP256V2LS lot 1	CAP256V2LS lot 2
3016.v5.c45	D	0.014	0.011	0.005	0.006
AC10.29	B	>50	>50	>50	>50
BG505.W6M.C2	A	0.027	0.014	0.005	0.006
CAP210.E8	C	0.016	0.013	0.006	0.008
CAP256.206.C9	C	0.007	0.005	0.002	0.001
CNE55	AE	0.026	0.016	0.006	0.007
KER2018.11	A	0.013	0.007	0.003	0.004
PVO.04	B	9.22	>50	>50	>50
T33-7	AG	>50	>50	>50	>50
ZM53.12	C	0.014	0.011	0.006	0.007

<0.001
.001-.01
.01-.100
.100-1.00
1.00-10.0
10-50
<50
µg/ml

Figure S3. CAP256V2LS preparations are equivalent, and are improved compared to laboratory stocks. A. Graphs show representative neutralization curves for selected Env-pseudoviruses by wild-type CAP256-VRC26.25 and K100mA mutant, both produced in HEK 293expi; and two lots of clinical grade product, produced in CHO cells. Lot 1 was used for 208 virus panel. Lot 2 is vialed product from 2000L production run. Graphs show mean and standard deviation of three independent experiments. **B.** IC50 and IC80 for the four antibodies, against the five Env-pseudoviruses in **A** plus 5 additional. Values are geometric means of three independent experiments. Lots 1 and 2 of CAP256V2LS are indistinguishable within the error of the assay.
